# New Remedies

**Published:** 1871-08

**Authors:** 


The first number of *New Remedies*, a quarterly retrospect of Therapeutics, Pharmacy, and allied subjects, has been received. It is edited by Horatio C. Wood, jr., M. D., of Philadelphia, and promises to be valuable to the profession. It is published by Wm. C. Wood & Co., New York.

